# Impact of atmospheric variables on incidence of acute coronary syndrome amongst Gujarati Asian Indians – IOAPIC study

**DOI:** 10.1016/j.ihj.2023.04.001

**Published:** 2023-04-06

**Authors:** Ashwati Konat, Kamal Sharma, Divya Chandel

**Affiliations:** a Department of Zoology, Biomedical Technology and Human Genetics, University School of Sciences, Gujarat University, Navrangpura, Ahmedabad, Gujarat, 380009, India; b SAL Hospital and Medical Institute, Doordarshan Tower, Opposite Drive In Road, Thaltej, Ahmedabad, Gujarat, 380054, India

**Keywords:** Seasonal epidemiology, Acute coronary syndrome, Humidity, Temperature, Atmospheric pressure

## Abstract

**Objectives:**

The study evaluated the effects of climatic conditions and its variables on incidence of ACS in Gujarati Asian Indians.

**Methods:**

In this multicentric, retrospective, observational case control study of a total of 3256 patients, the Electronic Medical Records (EMS) of all 740 patients who were hospitalised for ACS at the 2 tertiary care centres of Ahmedabad over 3 years between January 2017 to December 2019 were compared with demographically matched 2516 non- ACS, CAD controls were compared for the impact of climatic parameters viz. temperature, barometric pressure, humidity as reported by monthly averages by the state meteorological department for monthly incidence of ACS.

**Results:**

The highest number of ACS cases were observed during the month of September (N = 127; 27%) followed by August (N = 123; 26%). Higher humidity and decreasing atmospheric pressure were associated with highest occurrence of ACS in the state of Gujarat. ST elevation Myocardial Infarction (STEMI) was the most common type of ACS (N = 598; 80.8%). In ACS, the Coefficient of correlation for humidity was 0.712 (P = 0.009), while that for temperature was 0.506 (P = 0.093). Effect of atmospheric pressure was found to be significant with a negative Coefficient of correlation of −0.571 (P = 0.052). However amongst the controls, the coefficient of correlation for humidity was 0.062 (P = 0.722) and atmospheric pressure Correlation coefficient was 0.107 (P = 0.539) non-significant.

**Conclusions:**

The higher humidity/temperature and lower atmospheric pressure had a positive correlation with the incidence of ACS with highest incidence noted in August and September in the state of Gujarat.

## 1 Introduction

In India, the Coronary Artery Disease (CAD) affects about 30 million individuals in India having CAD.^1^ In 2004, WHO reported Cardiovascular Disease(CVD) death rates for all ages as 381.5 per 100,000 in India.^2^ A study established that the mean age of occurrence of STEMI in Indians is 5–10 years lower than the Western population.^3^ Previous studies suggest that there is a significant seasonality with respect to the incidence of Acute Coronary Syndrome (ACS).^4^^,^^5^ However, only a few studies have addressed the effect of barometric pressure on ACS.^6^^,^^7^ The term Acute Coronary Syndrome (ACS) refers to group of clinical symptoms compatible with Acute Myocardial Ischemia including Unstable Angina (UA), Non-ST-segment elevation Myocardial Infarction (NSTEMI), and ST-segment elevation Myocardial Infarction (STEMI).

Recent literature survey suggests that the environmental risk factors have emerged as a dominant public health concern with respect to the weather variations and its impact on human health especially in light of Global warming with studies showing that cardiovascular disease (CVD) is sensitive to the variability of weather throughout the world.^8^ With economic development and changes in dietary and lifestyle patterns, CVD deaths have increased and are predicted to become a major cause of morbidity and mortality by 2020.^9^

This study examined any possible relationship of the climatic conditions like temperature, atmospheric pressure and humidity with respect to the monthly incidence of ACS in the state of Gujarat. A detailed understanding of the seasonal pattern of ACS and the climatic parameters influence on its occurrence might aid in the preventive strategies both from the climate change as well as health prevention strategies.

## 2 Methods

In this multicentric, retrospective, observational case control study of a total of 3256 patients, the Electronic Medical Records (EMR) of all 740 patients who were hospitalised for ACS at the 2 tertiary care centres of Ahmedabad over 3 years between January 2017 to December 2019 were compared with demographically matched 2516 non- ACS, CAD controls for the impact of climatic parameters viz. temperature, barometric pressure, humidity as reported as the monthly averages by the state meteorological department for the monthly incidence of ACS. The meteorological data was recorded from the website of Indian meteorological department.

ACS was defined by ESC 2020^10^ guidelines as patients with acute chest pain and persistent (>20 min) ST-segment elevation. Patients with acute chest discomfort but no persistent ST-segment elevation [non-ST-segment elevation ACS (NSTEACS)] exhibit ECG changes that may include transient ST-segment elevation, persistent or transient ST-segment depression, T-wave inversion, flat T waves, or pseudonormalization of T waves; or the ECG may be normal.

The parameters analysed were**: a)** the type and frequency of ACS according to months. b) Demographic parameters such as age and gender. c) Monthly mean temperature, humidity and atmospheric pressure.

Inclusion criteria: Patients hospitalized with Coronary Artery Disease (CAD) either with ACS or Non-ACS CAD within the age group of 19–85 years old.

Exclusion criteria: Non hospitalized CAD patients, non-obstructive heart diseases, hypertension, other heart diseases and without angioplasty were excluded.

### 2.1 Statistical analysis

Statistical analysis was done using software SPSS v.21. Categorical variables were defined as percentage. The chi-square test and ANOVA test were used for the analysis of the categorical variables. A correlation analysis between the variables was determined by using Pearson's correlation test and statistical significance was accepted at the level of p < 0.05.

## 3 Results

From January 2017 to December 2019, 740 eligible patients with ACS and 2516 non ACS subjects were analysed in this study.

### 3.1 Age, sex distribution and types of ACS

The highest percentage for Mean Age for ACS for males were in the age group of 51–60 years (35.5% of total males) while the highest percent of females were in the age group of 51–60 (30.1% of total females. Out of the total 740 cases, 143(19.2%) were females while 592(80.8%) were males. Amongst types of ACS, a decade wise grouping of patients was done. Our study showed that STEMI was found to be more prevalent than NSTEMI followed by UA. STEMI was found to be maximum in the age group of 61–70 years [211(82.4%)] whereas NSTEMI was more in the age group 71–80 years (16.5%) and UA was more in the age group 61–70years [22 (8.6%)]. This difference was not statistically significant in our study (Table 1).

**Table 1:** Decade-wise number of cases of three years (2017,2018,2019) and types of ACS.

Sr.No	Age	Male (N (%))	Female(N (%))	P-value	STEMI(N (%))	NSTEMI(N (%))	UA(N (%))	P-value
1	<30	4(0.7)	2(1.4)	0.05	4(2.6)	1(0.6)		0.425
2	31–40	34(5.7)	2(1.4)		4(66.7)	1(16.7)	1(16.7)	
3	41–50	126(21.1)	31(21.7)		30(81.1)	5(13.5)	2(5.4)	
4	51–60	212(35.5)	43(30.1)		134(84.8)	16(10.1)	8(5.1)	
5	61–70	150(25.1)	42(29.4)		211(82.4)	23(9.4)	22(8.6)	
6	71–80	56(9.4)	20(14.0)		147(75.8)	32(116.5)	15(7.7)	
7	81–90	10(1.7)	3(2.1)		12(92.3)	1(7.7)		
	TOTAL	592	143					

ACS : Acute Coronary Syndrome, STEMI: ST Elevation Myocardial Infarction, NSTEMI: Non-ST Elevation Myocardial Infarction, UA: Unstable Angina, Correlation is significant at the 0.05 level.

### 3.2 Monthly incidence of ACS and control

In our study, the maximum numbers of cases were recorded in the month of September 108(18.1%) followed by August, 101 (16.9%). Higher number of females in our study presented with ACS in the month of August i.e., 22(15.4%) (Table 2), while the maximum number of control was in the month of July i.e., 250 (10%) (Table 3).

**Table 2:** Month wise number of cases for three years (2017,2018,2019).

Sr.No	Month	Male	%	Female	%	P-value
1	January	17	2.8	4	2.8	0.188
2	February	20	3.4	3	2.1	
3	March	29	4.9	13	9.1	
4	April	48	8	11	7.7	
5	May	51	8.5	17	11.9	
6	June	57	9.5	17	11.9	
7	July	61	10.2	8	5.6	
8	August	101	16.9	22	15.4	
9	September	108	18.1	19	13	
10	October	48	8	9	6.3	
11	November	27	4.5	12	8.4	
12	December	30	5	8	5.6	

Correlation is significant at the 0.05 level.

**Table 3:** Month wise number of cases vs control for three years (2017,2018,2019).

Sr.No	Month	Cases	%	Control	%	P-value
1	January	21	3	217	9	>0.05
2	February	23	3	189	8	
3	March	42	6	242	10	
4	April	59	8	206	8	
5	May	68	9	205	8	
6	June	74	10	204	8	
7	July	69	9	250	10	
8	August	123	17	221	9	
9	September	127	17	226	9	
10	October	57	8	178	7	
11	November	39	5	171	7	
12	December	38	5	207	8	

### 3.3 Meteorological parameters

A detailed incidence of type of ACS with different meteorological parameters has been recorded in (Table 4). The parameters considered were minimum temperature, maximum temperature, average temperature, humidity and atmospheric pressure.

**Table 4:** Types of ACS in different meteorological parameters.

Sr.No.	Variable	N(%)	STEMI	NSTEMI	UA	P-Value
1 Minimum Temperature (^o^C)						
15–20		62(8.4)	53(8.9)	5(5.6)	4(7.5)	0.000
21–25		73(9.9)	54(9.0)	4(4.5)	15(28.3)	
25–30		523(70.7)	424(70.9)	70(78.7)	29(54.7)	
30–35		19(2.6)	13(2.2)	1(1.1)	5(9.4)	
2 Maximum Temperature (^o^C)						
30–35		88(11.9)	59(9.9)	5(5.6)	24(45.3)	0.000
35–40		333(45.6)	267(44.6)	50(56.2)	16(30.2)	
40–45		129(17.4)	103(17.2)	13(14.6)	13(24.5)	
45–50		190(25.7)	169(28.3)	21(23.6)		
3 Average Temperature (^o^C)						
19–25		91(12.3)	74(12.4)	6(6.7)	11(20.8)	0.000
26–30		21(2.8)	12(2.0)	9(17.0)		
31–35		296(40.0)	257(43.0)	34(38.2)	5(9.4)	
35–40		6(0.8)	5(0.8)	1(1.1)	25(47.2)	
4 Humidity (%)						
25–30		98(13.1)	78(13)	12(13.5)	8(15.1)	0.000
30–35		3(0.4)	3(0.5)			
35–40		82(11.0)	79(13.2)	3(3.4)		
40–45		61(8.2)	64(10.7)	11(12.4)		
45–50		48(6.4)	41(6.9)	5(5.6)	2(3.8)	
50–55		31(4.2)	22(O3.7)		9(17.0)	
55–60		69(9.2)	55(9.2)	9(10.1)	5(9.4)	
60–65		30(4.0)	24(4.0)	6(11.3)		
65–70		132(17.7)	104(17.4)	20(22.5)	8(15.1)	
70–75		6(0.8)	5(0.8)	1(1.9)		
75–80		19(2.5)	12(2.0)	2(2.2)	5(9.4)	
80–85		56(7.5)	47(7.9)	6(6.7)	3(5.7)	
85–90		91(12.2)	64(10.7)	21(23.6)	6(11.3)	
5 Atmospheric Pressure (mbar)						
980–990		76(10.2)	64(10.7)	9(10.1)	3(5.7)	0.000
990–1000		293(39.3)	237(39.6)	39(43.8)	17(32.1)	
1000–1010		344(46.1)	274(45.8)	37(41.6)	33(62.3)	
1010–1020		13(1.7)	13(2.2)	85(95.5)		

ACS :Acute Coronary Syndrome, STEMI: ST Elevation Myocardial Infarction, NSTEMI: Non-ST Elevation Myocardial Infarction, UA: Unstable Angina, Correlation is significant at the 0.05 level.

### 3.4 Atmospheric pressure

**Fig. 1:**
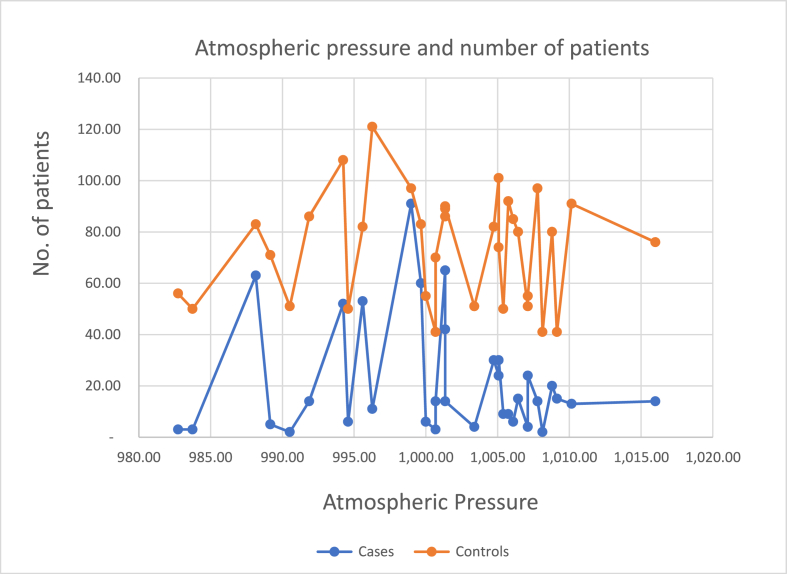
Number of patients vs Atmospheric pressure.

Gujarat does not experience huge variation in atmospheric pressure. In the month of April, May, June, July, August, September, it has lower atmospheric pressure as compared to the winter months of November, December, January, February and March which have higher atmospheric pressure. Fig. 1 shows the distribution of ACS and Non ACS, CAD patients across various atmospheric pressure.

### 3.5 Temperature

**Fig. 2:**
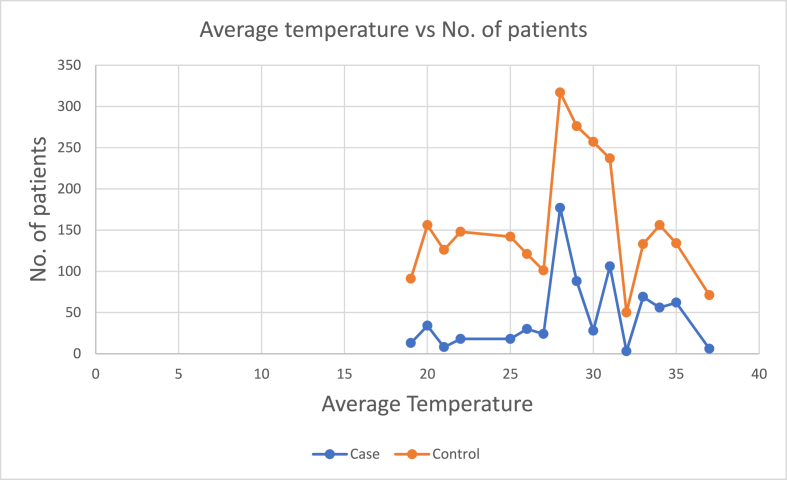
Number of patients vs Average Temperature.

Gujarat does not go through extreme cold temperature and the average winter temperature of Gujarat varies between 15^0^C–37 °C. In summer, temperatures are higher, fluctuating from 25 °C in night to 47 °C in day time. In the monsoon season, the day time temperature is around 41 °C and night temperature is around 26 °C. In our study, we found that the highest temperature was recorded in the month of May and lowest in January. A graph representing the distribution of ACS and Non ACS CAD patients during various average temperature has been included Fig. 2.

### 3.6 Humidity

**Fig. 3:**
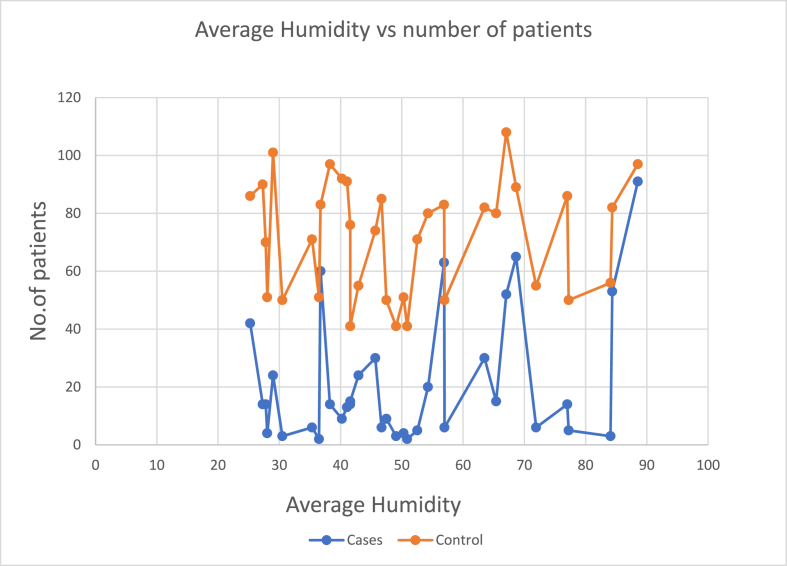
Number of patients vs Humidity.

Since Gujarat is the coastal area of India it has higher humidity, with the lowest being in the month of April and March (29% and 31% respectively), and highest being in the month of August and September (85% and 89% respectively). Humidity in the months of December, January and February are near to the comfortable zone of humidity (57%, 44% and 42% respectively). In the current study we have found that the number of patients are maximum in the months having relatively high humidity. Fig. 3 represents the number of ACS and Non ACS CAD patients during different humidity level.

### 3.7 Season – wise distribution of ACS

The months were divided into three categories, Monsoon (July to October), Summer (March to June) and Winter (November to February). The maximum number of cases were found in Monsoon season with STEMI 311(79.1%), followed by NSTEMI with 62(15.8%) cases, followed by UA with 20 (5.1%) cases. During Summer STEMI (168; 87.5%) was more compared to NSTEMI (16; 8.3%) and UA (8; 4.2%). While in Winter, STEMI (119; 76.8%) and UA (25; 16.15%) were found to be more than NSTEMI (11; 7.1%). Our study has revealed that the highest number of ACS cases were recorded during the monsoon season (Table 5).

**Table 5:** Season wise types of ACS.

Sr.No	Variable	STEMI	NSTEMI	UA	P-Value
1	Monsoon (July to October)	311(79.1)	62(15.8)	20(5.1)	0.001
2	Summer (March to June)	168(87.5)	16(8.3)	8(4.2)	
3	Winter (November to February)	119(76.8)	11(7.1)	25(16.1)	

ACS = Acute Coronary Syndrome, STEMI: ST Elevation Myocardial Infarction, NSTEMI: Non-ST Elevation Myocardial Infarction, UA: Unstable Angina, Correlation is significant at the 0.05 level.

For the incidence of ACS, the Coefficient of correlation for humidity was 0.712 (P = 0.009), while that for temperature was 0.506 (P = 0.093). Effect of atmospheric pressure was found to be significant with a negative Coefficient of correlation of −0.571 (P = 0.052) (Table 6).

**Table 6:** Correlation Analysis of Metereological parameters with Cases.

Variables	Month	Avg no.of cases	Avg. Temperature	Avg. Humidity	Avg. Atm Pressure
Month					
Correlation					
Coefficient	1	0.343	−0.028	0.547	0.089
Sig(2-tailed)		0.275	0.931	0.065	0.784
Avg no. of cases					
Correlation					
Coefficient	0.343	1	0.506	0.712	−0.571
Sig(2-tailed)	0.275		0.093	0.009^a^	0.052^a^
Avg. Temperature					
Correlation					
Coefficient	−0.028	−0.01	1	−0.012	−0.768
Sig(2-tailed)	0.931	0.506		0.971	0.004
Avg. Humidity					
Correlation					
Coefficient	0.547	0.712	−0.012	1	−0.342
Sig(2-tailed)	0.065	0.009^a^	0.971		0.277
Avg. Atm. Pressure					
Correlation					
Coefficient	0.089	−0.571	−0.768	−0.342	1
Sig(2-tailed)	0.784	0.052^a^	0.004	0.277	

a Correlation is significant at the 0.05 level (2-tailed), Avg: Average, Atm: Atmospheric.

However amongst the controls, the coefficient of correlation for humidity was 0.062 (P = 0.722) and atmospheric pressure Correlation coefficient was 0.107 (P = 0.539) which was found to be non-significant (Table 7).

**Table 7:** Correlation Analysis of Metereological parameters with Case vs Control.

Variables	Case	Control	Avg. Temperature	Avg. Humidity	Avg. Atm Pressure
Case					
Correlation					
Coefficient	1	0.579	0.217	0.330	−0.070
Sig(2-tailed)		0.000^a^	0.210	0.05^a^	0.689
Control					
Correlation					
Coefficient	0.579	1	−0.099	0.062	0.107
Sig(2-tailed)	0.000^a^		0.572	0.722	0.539
Avg. Temperature					
Correlation					
Coefficient	0.217	−0.099	1	−0.105	−0.638
Sig(2-tailed)	0.210	0.572		0.549	0.000^a^
Avg. Humidity					
Correlation					
Coefficient	0.330	0.062	−0.105	1	−0.342
Sig(2-tailed)	0.05^a^	0.722	0.549		0.076
Avg. Atm. Pressure					
Correlation					
Coefficient	−0.070	0.107	−0.638	−0.304	1
Sig(2-tailed)	0.689	0.539	0.000^a^	0.076	

a Correlation is significant at the 0.05 level (2-tailed), Avg: Average, Atm: Atmospheric.

## 4 Discussion

Gujarat lies in the western part of India that carries a vast coastline. With the land closer to the coasts tend to be humid whereas the areas lying in the centre of the state experience a completely diverse climatic condition.

In our study we have found that humidity and atmospheric pressure have shown an increased occurrence of ACS. Our study shows that highest cases of Acute Coronary Syndrome were recorded in the month of September, where humidity and low atmospheric pressure were the major correlates in the Gujarat region. Raised humidity decreases the body's efficiency in dissipating the metabolic heat, while low humidity can lead to dehydration. The first adaptive mechanism to maintain a healthy core temperature under heat stress is by sweating, but humid conditions can interfere and prevent from effectively managing the core body temperature thus impacting not only cellular metabolic needs but myocardial energetics as well. Raised sweating increases the risk of dehydration as it lowers the amount of fluid in the body causing additional strain on the myocardium. STEMI as a well-recognized manifestation of severe coronary artery spasm due to dehydration has been documented.^11^ Individuals with underlying cardiovascular disease can have deleterious effects and in some cases can lead to thrombosis. There have been studies showing a positive correlation between the number of cardiovascular disease deaths and relative humidity.^12^ The higher humidity is known to increase risk of ACS because of physiological responses of heat dispersion being curtailed.

The study findings suggests an association of lower atmospheric pressure and incidence of ACS as well. These findings are also similar with Danet et al,^13^ who found a 10-mbar decrease <1016 mbar and a 10-mbar increase >1016 mbar to be associated with a 12% increase (*P* = 0.001) and an 11% increase (*P* = 0.01) in event rates, respectively. These effects were independent and influenced both coronary morbidity and mortality rates, with stronger effects in older age groups and for recurrent events. Houck et al^14^ performed a Logistic regression analysis to correlate the presence or absence of AMI to the maximum difference in pressure per 24-h period and the maximum rate of change in atmospheric pressure per 24-h period. There was a significant correlation (p = 0.0083) in the rate of pressure change and the occurrence of AMI (odds ratio 1.115). In other words, for a 1.0 IH/hour pressure decrease, the odds of having ≥1 AMI on the following day increased by approximately 10%.^14^

Several studies have reported the occurrence of Acute Myocardial Infarction to be associated with lowee temperatures. Winter surge in the number of ACS cases was observed in an older study of acute myocardial infarction amongst the Italy population,^15^ as well in the HELIOS study which showed increase in the cases in the winter.^16^ In a study of 64,191 cases with ACS in Italy, the admissions were more in the month of January.^15^

The study by Dilaveris et al^16^ indicated that as temperature increases, the number of daily deaths caused by Acute Myocardial Infarction (AMI) declines, reaching a trough corresponding to 23.3 °C, then begins to rise as the temperature rises.^17^

Some studies have shown that the combined effect of humidity with cold is additive. In Cyprus the highest cases were observed in the month of January when it was having 87% humidity and average temperature were 6 °C.^18^ In Greece, the highest cases were in January when the humidity was highest around 70% with lower temperature of 7 °C.^19^ In Innsbruck where, warm winter average temperature was 12 °C–23 °C and cold winter was from 0 °C to −5 °C, the number of cases were more in cold winter than in warm winter.^5^

In our study, we noticed a stronger association between increased humidity and lower atmospheric pressure (<1010) with higher rates of ACS compared to the more commonly studied parameters such as temperature.^6^^,^^15^ The mean age was lower in men with ACS than in women (men: 62.2 ± 12 years vs. women: 70.2 ± 12 years). These findings are similar to those of the Greek HELIOS study where the mean age of men was 65 ± 12 years and of women 74 ± 10 years.^19^

The most affected age group in men was between 61 and 70 years whereas females were from 71 to 80 years age group. The ageing induces deterioration in the thermoregulation and homeostasis which along with the increasing prevalence of the chronic conditions, enhances vulnerability to both cold and heat in elderly. These results and the age difference in our study are similar to countries like China. It has been observed that Gujarati Asian Indians are ‘older’ for their vascular age by 6.54 (9.5) years when compared with their chronological age due to higher prevalence of dyslipidaemia, hypertension and centripetal obesity, apart from chronological ageing and male gender.^20^

In our cohort, 80.8% of patients were having STEMI type of ACS, 12% were having NSTEMI of ACS and only 7.2% were having unstable angina. The proportion of STEMI among all ethnic groups (74%) was higher compared to earlier ACS registries in developed countries including registries like GRACE.^21^ Higher proportion of STEMI compared to NSTEMI and UA in NCVD was similar to those in the CREATE registry (47.3–71.4%).^22^

Higuma et al^23^ found there was no significant association of age and sex with temperature and humidity on AMI hospitalization, but differed by season. However, higher temperatures in spring and higher humidity in autumn were risk factors for AMI hospitalization.

The study has implications in terms of implementation of climate change policies globally not only from the perspective of infectious disease but also from the perspective of CVD. The study can help also to prognosticate and plan health care policies in CVD implementation with regards to atmospheric variables.

### 4.1 Study limitations

Firstly, This study represents only datasets from ACS cases and atmospheric variables from the state of Gujarat only. Even though we have 740 ACS cases and 2515 non ACS CAD controls, we propose that a future larger multicentric prospective study with a bigger sample size across the country as well as from the world may yield better results. We hope that this study shall pave the way ahead and lay foundation of similar studies in the future. Secondly, the non availability of moving averages of the atmospheric variables viz. humidity, atmospheric pressure and temperature for various cities from where the patients came has limited our analysis to the available monthly averages of these parameters.

## 5 Conclusion

In this study we observed that the maximum number of ACS cases were observed in month of September and August. The higher humidity with an average temperature showed a significant relationship with ACS in Gujarati Asian Indians. STEMI was the most common type of ACS which occurred in this population followed by NSTEMI and unstable angina. The average temperature of (30–35 °C) with humidity (65–70%) was associated with the highest incidence of ACS in our population subgroup. However there was no correlation of these atmospheric variables on non ACS CAD patients hospitalized during the study period.

## Funding

This research did not receive any specific grant from funding agencies in the public, commercial, or not - for -profit sectors.

## Declaration of competing interest

The authors declare that they have no known competing financial interests or personal relationships that could have appeared to influence the work reported in this paper.
